# The impact of COVID-19 related adversity on the course of mental health during the pandemic and the role of protective factors: a longitudinal study among older adults in The Netherlands

**DOI:** 10.1007/s00127-023-02457-5

**Published:** 2023-03-25

**Authors:** Tjalling J. Holwerda, Eva Jaarsma, Elisabeth M. van Zutphen, Aartjan T. F. Beekman, Kuan-Yu Pan, Majogé van Vliet, Najada Stringa, Judith H. van den Besselaar, Janet L. MacNeil-Vroomen, Emiel O. Hoogendijk, Almar A. L. Kok

**Affiliations:** 1 Department of Epidemiology & Data Science, Amsterdam Public Health Research Institute, Amsterdam UMC Location Vrije Universiteit, De Boelelaan 1117, Amsterdam, The Netherlands; 2 Department of Psychiatry, ARKIN Mental Health Care Amsterdam, Amsterdam, The Netherlands; 3 Department of Psychiatry, Amsterdam Public Health Research Institute, Amsterdam UMC Location Vrije Universiteit, De Boelelaan 1117, Amsterdam, The Netherlands; 4 Department of Internal Medicine, Amsterdam Public Health Research Institute, Amsterdam UMC Location University of Amsterdam, Meibergdreef 9, Amsterdam, The Netherlands; 5 Amsterdam UMC Location Vrije Universiteit, Van der Boechorststraat 7, 1081 BT Amsterdam, The Netherlands

**Keywords:** COVID-19, Anxiety, Depression, Loneliness, Resilience, Mental health

## Abstract

**Purpose:**

Many studies report about risk factors associated with adverse changes in mental health during the COVID-19 pandemic while few studies report about protective and buffering factors, especially in older adults. We present an observational study to assess protective and buffering factors against COVID-19 related adverse mental health changes in older adults.

**Methods:**

899 older adults (55 +) in the Netherlands were followed from 2018/19 to two pandemic time points (June–October 2020 and March–August 2021). Questionnaires included exposure to pandemic-related adversities (“COVID-19 exposure”), depressive and anxiety symptoms, loneliness, and pre-pandemic functioning. Linear regression analyses estimated main effects of COVID-19 exposure and protective factors on mental health changes; interaction effects were tested to identify buffering factors.

**Results:**

Compared to pre-pandemic, anxiety symptoms, depression symptoms and loneliness increased. A higher score on the COVID-19 adversity index was associated with stronger negative mental health changes. Main effects: internet use and high mastery decreased depressive symptoms; a larger network decreased anxiety symptoms; female gender, larger network size and praying decreased loneliness. COVID-19 vaccination buffered against COVID-19 exposure-induced anxiety and loneliness, a partner buffered against COVID-19 exposure induced loneliness.

**Conclusion:**

Exposure to COVID-19 adversity had a cumulative negative impact on mental health. Improving coping, finding meaning, stimulating existing religious and spiritual resources, network interventions and stimulating internet use may enable older adults to maintain mental health during events with large societal impact, yet these factors appear protective regardless of exposure to specific adversities. COVID-19 vaccination had a positive effect on mental health.

**Supplementary Information:**

The online version contains supplementary material available at 10.1007/s00127-023-02457-5.

## Introduction

The ongoing COVID-19 pandemic has caused worldwide disruptions in the lives of people. Current evidence suggests that the pandemic has an important impact on mental health and loneliness in the general population and in specific groups such as people with pre-existing mental illness, either directly, through the physical impact of the virus, or indirectly, through uncertainty, social isolation, or quarantine [1, 2]. Older adults have a higher risk of physical disease, complications, and excess mortality due to COVID-19 infection and face increased mental health risks due to economic, social and psychological consequences of the pandemic [3–6]. Research on older adults is of particular concern because they are at risk of exacerbation of existing isolation, loneliness, medical comorbidities, and negative life events such as the death of loved ones during an already potentially burdensome period [7, 8] Indeed, the available evidence so far showed that in older adults, psychological distress, loneliness, anxiety, and depressive symptoms were higher in the first months of the pandemic compared to their pre-pandemic levels [8, 9]. However, the rapidly growing evidence on the mental health consequences of COVID-19 is fragmented and often limited due to use of probability and convenience samples [10], with few studies on older adults having data on pre-pandemic functioning. Moreover, few studies examine longer-term mental health outcomes beyond the first months of the pandemic. Therefore, longitudinal data covering pre-pandemic and pandemic time points beyond the first months are necessary to study how the COVID-19 pandemic affects the course of mental health in older adults.

From a perspective of resilience, the COVID-19 pandemic is a large challenge given the variety of disruptions and stressors that accompany it. Most resilience theorists agree that resilience is the result of active, dynamic adaptation to stressful circumstances [11]. The COVID-19 pandemic has caused acute stress due to the presence of a life-threatening disease, lockdown measures and physical distancing and ongoing stress causing longer term consequences, such as psychological and societal effects. Therefore, the pandemic requires adaptation efforts on several levels and time scales. Yet the extent to which this adaptation is needed, may depend strongly on the extent to which an individual is actually exposed to adversity. Many previous studies implicitly regarded the COVID-19 pandemic as a homogeneous exposure to all older adults but did not examine how older adults vary in their actual exposure to concrete adverse situations related to the pandemic, such as having been in quarantine or losing a loved one to COVID-19.

Furthermore, most studies have focused only on ‘main effects’ of demographic and psychosocial factors on mental health, i.e., whether these factors are on average related with changes in mental health from before to during the pandemic. Previous studies suggested internet use for interpersonal communication, and found lower subjective age, proactive coping and greater self-reported psychological resilience, to protect against the impact of COVID-19 [4, 12–15]. Previous work showed that during the first six months of the COVID-19 pandemic, loneliness increased for almost all older adults, and that having a partner, high mastery and good physical functioning were associated with less increase in loneliness [16]. However, beyond ‘main effects’, we are not aware of studies that examined potential ‘buffering effects’ of physical, social and psychological resources, i.e. whether these factors can help to reduce the impact of COVID-19 exposures on mental health.

Attending to these gaps in knowledge on mental health resilience to the COVID-19 pandemic in older adults, our study examined whether the impact of individual-level exposure to COVID-19-related adversities on mental health was ameliorated by specific protective factors. These factors include physical health, interpersonal relationships, demographic and psychological characteristics, accessibility and continuity of care as well as financial factors.

Our study had the following research questions:

1. What was the course of depressive symptoms, anxiety symptoms and loneliness in older adults across a pre-pandemic time point (2018/19) and two COVID-19 pandemic time points (in 2020 and 2021)?
2. What was the association between COVID-19 related adversity measured by means of a COVID-19 exposure index [17] and changes in depressive symptomatology, anxiety symptoms and loneliness in older adults over time?
3. Main effects: which physical, social, lifestyle and psychological factors are associated with these changes in mental health, net of COVID-19 exposure?
4. Buffering effects: which of these factors help to reduce the impact of COVID-19 related adversity on changes in mental health?

## Methods

### Study design, participants, and procedures

**Fig. 1:**
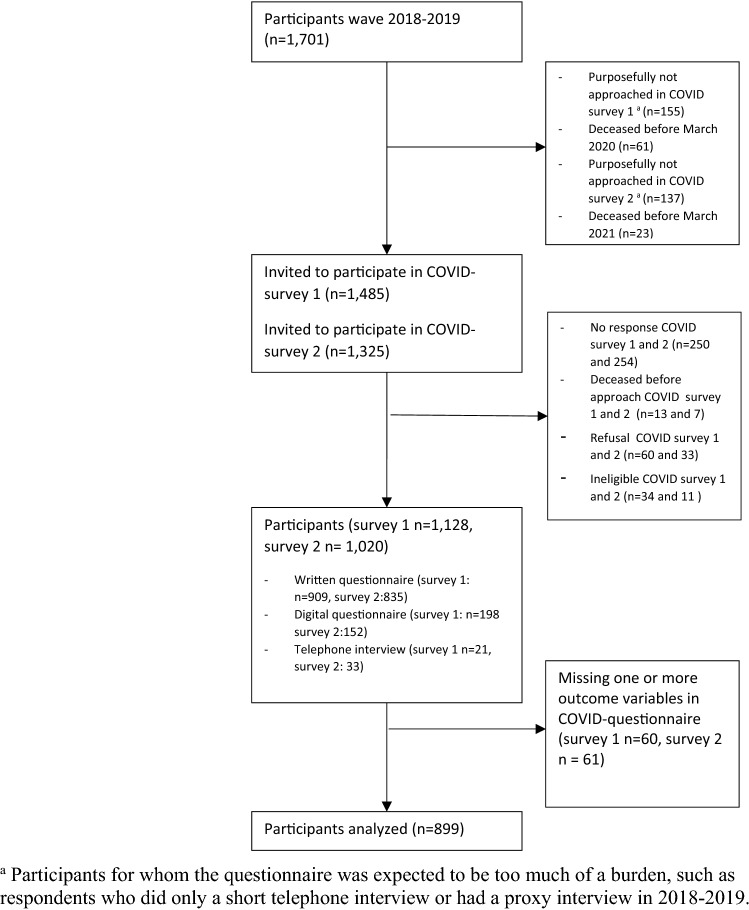
Flow diagram on analysis of depressive symptoms, anxiety symptoms and loneliness of the Longitudinal Aging Study Amsterdam (2018–2019, 2020, 2021)

LASA is a population-based cohort study with data from 1992 onwards in older adults aged 55–84 years (n = 3107) in the Netherlands. Participants were interviewed three times per ten years. In 2002 (n = 1002) and 2012 (n = 1023) two refresher cohorts aged 55–64 years were added. LASA was approved by the institutional review board of the VU University Medical Centre. Informed consent was obtained from all participants [18, 19]. Of the 1701 participants of the last pre-pandemic measurement (2018–2019), n = 1485 were selected for the first COVID-19 survey, sent in June 2020; reminders were sent in July 2020. Participants not selected had deceased (n = 61) or the survey was considered too much of a burden for them (n = 155). Questionnaires were sent by postal mail, which participants could return by mail, or fill in digitally. Participants from the first cohort, aged 80 years and older, initially not responding were offered to answer the questionnaire in a telephone interview. Of the 1485 participants approached, 1128 (76%) returned the first COVID-19 questionnaire. In March 2021, a second questionnaire with additional telephone option was sent to 1325 participants (137 participants refused / were not able to participate, 23 had deceased). Reminders were sent in May 2021. N = 1020 (69%) returned the second questionnaire. After excluding participants with missing data on anxiety, depression, and loneliness the analytic sample consisted of 899 participants (Fig. 1).

## Measures

### COVID-19 cumulative exposure

We used a COVID-19 exposure index developed, tested and validated in previous research [17]. In brief, we adopted a methodology from the frailty research field. In a frailty index, individual deficits indicative of frailty are combined; where a score of 0 means no deficits and a score of 1 means all deficits are present. The index score is calculated by dividing the sum of items by the number of items [20–22]. Likewise, to operationalize exposure to pandemic-related adversity, a 35-item COVID-19 exposure index was developed based on direct and indirect exposures to the COVID-19 pandemic. These included COVID-19 infection of participants and their relatives, consequences of COVID-19 infection, of social restrictions and pandemic-related pressure on the health care system. For a full overview of items, see supplementary file 1. For each item, participants scored 0 (no) or 1 (yes). We calculated a cumulative exposure index based on the two COVID-19 measurements. If a participant tested positive for COVID-19, was admitted to hospital or intensive care unit due to COVID-19, their score on the respective item would be 2, regardless of whether they reported this once or twice as we considered COVID-19 disease as having more impact than the other items and it was less likely to be reported twice compared to other items. For the remaining items, we calculated the sum score of the items (0, 1 or 2). Next, we summed all scores, resulting in a combined COVID-exposure index with a range of 0–70 [17]. Although this index was developed to use among Dutch older adults, the approach could be of use in general and non-Western populations.

### Mental health outcomes

Depressive symptoms were assessed with the Centre for Epidemiologic Studies Depression scale (CES-D, short version, 10-item scale). This self-report questionnaire measuring depressive symptoms in the general population has good psychometric properties and validity in older populations. The sum score ranges from zero to 30, with higher scores indicating more severe depressive symptoms. For the 10-item variant a cut-off score of  ≥ 10 is used to determine a probable depression [23–25]

Anxiety symptoms were measured with the Hospital Anxiety Depression Scale—Anxiety subscale (HADS-A) [26]. The HADS-A subscale consists of seven items for measuring anxiety. The sum score ranges from zero to 21, with higher scores indicating more severe anxiety. The HADS-A has a cut-off of eight or higher to indicate clinically relevant anxiety.

Loneliness was assessed using the 11-item De Jong Gierveld scale [27]. Loneliness scores range from zero to 11, higher scores indicate more severe loneliness. A score of three or higher indicates loneliness.

In the statistical models the three mental health outcomes were used as continuous variables.

### Potential protective factors

Following literature on resilience in diverse contexts, we included a range of potential protective factors that cover somatic, lifestyle, social, psychological, and socioeconomic domains [28, 29].

Functional limitations were assessed by seven activities: going up and down the stairs 15 steps without stopping, using public transportation on their own, cutting own toenails, getting (un)dressed, sitting down, and standing up from a chair, walking outside for five minutes without stopping, taking shower or bath on their own. Participants could answer on a 5-point Likert scale (without difficulty, with some difficulty, with much difficulty, only with help, cannot). Participants that reported at least some difficulties were considered limited for the activity. The number of activities with limitations was counted (number of items with at least some difficulty or worse, range zero to seven).

COVID-19 vaccination status was assessed with the question if the participant was vaccinated (no/yes) in the 2021 survey.

Frequency of praying/meditating was measured with a seven-point Likert scale (never, less than once a month/once a month, a few times a month, once a week, a few times a week, once a day, multiple times a day). In the analyses praying was categorized into never, up to once a day and more than once a day.

The personality trait of neuroticism, or emotional stability, was measured with an abbreviated (15 items) version of the NPV, the Nederlandse Persoonlijkheids Vragenlijst (DPQ, the Dutch Personality Questionnaire). Higher scores indicate less emotional stability [30].

Mastery was measured with the seven-item Pearlin Mastery Scale [31]. Sum scores could range from seven to 35. Higher scores indicate a stronger internal locus of control reflecting the perception that events in one's life relate to one's own actions rather than to external sources like powerful other persons, institutions, or circumstances.

Partner status was measured with the question if the participant had a partner (no/yes).

Network size was defined as the number of network members (≥ 18 years) with whom the participant had important/frequent contact and measured with a network delineation methodology described in more detail by Cochran et al. [32]. Network size ranged from zero to 79.

Internet use for social contact was measured with the question if the participant used internet to keep contact with other people (no/yes). Participants who did not have internet access or a device were categorized non-users.

Highest completed educational level was asked in nine categories, which were recoded to the nominal number of years it takes to complete that level (range 5–18 years).

Monthly household income was measured in 25 categories, ranging from 0–453 euros (lowest income category) to 5446 or more euros (highest income category). We recoded the categories to their median values, divided the median values by 500 and used this as a continuous variable.

All potential protective factors, except COVID-19 vaccination status, were available in the last regular LASA measurement. In the two COVID-19 surveys protective factors were not assessed.

### Statistical analyses

Baseline characteristics are presented as means with their standard deviation for continuous variables and as percentages for categorical variables. Analyses were conducted separately for depressive symptoms, anxiety symptoms and loneliness.

To answer the first research question, we used paired t-tests to describe and test average changes in depressive symptomatology, anxious symptomatology, and loneliness between the three included time points (i.e., the pre-pandemic time point (2018/19), and the first and second COVID-19 time points).

To answer the second research question, we estimated the effect of cumulative COVID-19 exposure on change scores of depressive symptoms, anxiety symptoms and loneliness using linear regression (model 1). Because our study focused on cumulative COVID-19 exposure and longer-term changes in mental health, in this analysis we used change scores between the pre-pandemic time point and the second COVID-19 time point. Positive change scores indicated worsening of mental health and negative scores indicated improvement. In model 2, we adjusted for pre-pandemic mental health.

To answer the third research question, we estimated main effects of protective factors adjusted for pre-pandemic mental health and all other protective factors (model 3). Unstandardized B’ s are shown for clinical interpretation and standardised B’s are shown to enable comparing outcomes (significance p < 0.05).

To answer our fourth research question, we examined buffering effects of protective factors. For this, we included a COVID-19 exposure index-by-protective factor interaction term, in separate models for each protective factor. If the interaction term indicated that the expected association between COVID-19 exposure and change in mental health was weaker in persons in whom the protective factor was present, this was interpreted as evidence for a buffering effect (significance p < 0.10) [33].

For the second, third and fourth research question we performed (nonhierarchical) multiple linear regression analysis.There were missing data on at least one of the potential protective factors or the exposure indices in 13.5% of the participants: functional limitations (0.2%), COVID-19 vaccination status (1.0%), internet use (1.1%), mastery (1.3%), network size (1.4%), COVID-19 exposure index at the second COVID-19 questionnaire (1.7%) and first COVID questionnaire (3.1%), neuroticism (3.3%) and income (4.6%). Missing data were handled using multiple imputation (predictive mean matching, 100 iterations, 14 imputations). Because imputing all items for COVID-19 exposure indices directly was not feasible, we used passive imputation; in our imputation model, the items of each exposure index were predicted by the other items from that exposure index, the total score of the other exposure index and all covariates, potential protective factors, and outcomes. We did not impute missing data on depressive symptoms, anxiety symptoms or loneliness as these were the outcomes of our study but included these as auxiliary variables in the imputation model. Analyses were conducted with IBM SPSS version 26.0 and the mice package in R version 4.0.3 [34].

## Results

### Descriptive statistics

Table 1 shows characteristics of participants with data on both COVID-19 time points, including demographics and potential protective factors. The 899 participants had a mean age of 72.3 years, 48.3% were male and 74.6% had a partner. The mean cumulative COVID-19 exposure index was 14.5 (standard deviation 6.2; observed range 0–34), indicating that there was substantial heterogeneity in COVID-19 exposure.

**Table 1:** Characteristics of the study population with available data on both time points, N = 899

	Mean or **%**	Standard deviation or n
Age, years, mean, sd	72.3	7.0
Sex, female, %, n	**51.7**	*465*
Cumulative covid exposure index, mean, sd	14.5	6.2
Potential protective factors		
Functional limitations, mean, sd	1.3	1.7
Vaccinated, %, n	**94.5**	*850*
Praying or meditating, %, n		
Never	**46.3**	*416*
Up to once a day	**35.9**	*323*
More than once a day	**17.8**	*160*
Neuroticism, mean, sd	4.5	5.0
Mastery, mean, sd	25.0	3.9
Partner, yes, %, n	**74.6**	671
Network size, mean, sd	17.8	10.2
Internet use, yes, %, n	**84.9**	*763*
Education, years, mean sd	11.6	3.4
Net monthly household income (€), mean, sd	2744.0	1160.8

Percentages are presented **bold**, n is presented *skewed*

### Prevalence of specific COVID-19 exposures

Over the two COVID-19 time points, 62 participants (6.6%) tested positive for COVID-19 and 8 (0.8%) were admitted to hospital for COVID-19. Of participants 129 (11.4%) had a first-degree family member with a positive COVID-19 test; 7 participants (0.6%) had a partner, parent or child with COVID-19 hospital admission or COVID-19-related death; 69 (6.1%) had a sibling, grandchild or other family member with COVID-19 hospital admission or COVID-19-related mortality and 236 (20.9%) had a neighbor, friend or other acquaintance with COVID-19-related hospital admission or death. Supplementary table 2 shows an overview of the prevalence of the specific COVID-19 exposures that were studied.

### Course of mental health during the pandemic

**Fig. 2:**
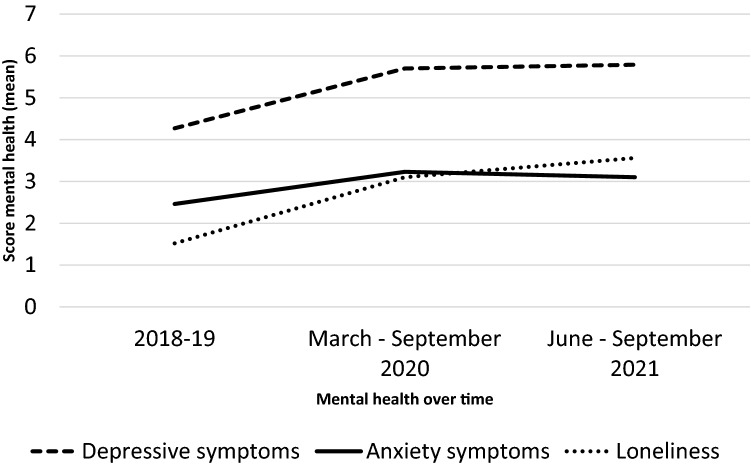
Mental health before and during the pandemic

On average, depressive symptoms, anxiety symptoms and loneliness were statistically significantly higher at the first COVID–19 time point (COVID 1) compared to the pre-pandemic time point (mean change scores 1.46, 0.77 and 1.60 respectively, all p values < 0.01). At the second COVID-19 time point (COVID 2) we found further increase in loneliness compared to COVID 1 (mean change score 0.44, p < 0.01) and stabilization of anxiety (mean change score 0.09, p = 0.41) and depressive symptomatology (mean change score –0.13, p = 0.13). Over the whole period, from the pre-pandemic time point to COVID 2, depressive symptoms, anxiety symptoms and loneliness increased (mean change scores were 1.53, 0.44 and 2.04 (all p-values < 0.01)). Results are shown in Fig. 2. An overview of change scores is shown in supplementary table 3.

### COVID-19 exposure and changes in mental health

Table 2 shows the results for changes in mental health due to COVID-19 exposure (model 1), adjusted for pre-pandemic mental health (model 2) and main effects of protective factors adjusted for pre-pandemic mental health and other protective factors (model 3).

**Table 2:** Main effects of predictors on change in mental health between 2018/19 and 2021

	Depression				Anxiety				Loneliness			
	Unst.B	CI	Std. B	p	Unst.B	CI	Std. B	p	Unst.B	CI	Std. B	p
Crude model (model 1)												
Covid-exposure	0.03	− 0.00 to 0.07	0.06	0.07	**0.04**	**0.02 to 0.7**	**0.11**	** < 0.01**	**0.10**	**0.08–0.13**	**0.28**	** < 0.01**
Adjusted for baseline mental health (model 2)												
Covid-exposure	**0.11**	**0.07 to 0.14**	**0.20**	** < 0.01**	**0.08**	**0.06 to 0.10**	**0.21**	** < 0.01**	**0.11**	**0.09–0.13**	**0.30**	** < 0.01**
Pre-covid mental health	− **0.41**	− **0.46 to** – **0.35**	− **0.48**	** < 0.01**	− **0.43**	− **0.49 to** – **0.37**	− **0.47**	** < 0.01**	− **0.26**	− **0.33 to** – **0.20**	− **0.26**	** < 0.01**
Model with all main effects (model 3)												
Covid-exposure	**0.11**	**0.08 to 0.14**	**0.21**	** < 0.01**	**0.08**	**0.06 to 0.1**	**0.20**	** < 0.01**	**0.12**	**0.10–0.14**	**0.32**	** < 0.01**
Pre-covid mental health	− **0.56**	− **0.63 to** – **0.50**	− **0.67**	** < 0.01**	− **0.56**	− **0.62 to** – **0.50**	− **0.61**	** < 0.01**	− **0.33**	− **0.40 to** − **0.26**	− **0.33**	** < 0.01**
Age	0.02	− 0.01 to 0.06	0.05	0.18	0.01	− 0.01 to 0.04	0.04	0.37	**0.03**	**0.01–0.06**	**0.10**	**0.02**
Sex (female)	0.04	− 0.37 to 0.45	0.01	0.85	0.22	− 0.09 to 0.52	0.09	0.17	− **0.33**	− **0.63 to** − **0.02**	− **0.14**	**0.04**
Education	0.04	− 0.03 to 0.10	0.04	0.24	0.02	− 0.03 to 0.07	0.03	0.41	0.01	− 0.04 to 0.05	0.01	0.84
Internet use	** − 0.79**	− **1.37 to 0.22**	− **0.24**	**0.01**	− 0.26	− 0.69 to 0.17	− 0.11	0.23	− 0.15	− **0.59 to 0.29**	− **0.06**	**0.51**
Network size	− 0.01	− 0.03 to 0.01	− 0.03	0.31	− **0.01**	− **0.03 to 0.00**	− **0.06**	**0.05**	− 0.03	− **0.04 to;** − **0.01**	− **0.12**	** < 0.01**
Neuroticism	**0.14**	**0.10 to 0.18**	**0.21**	** < 0.01**	**0.12**	**0.09 to 0.16**	**0.25**	** < 0.01**	0.05	0.02–0.08	0.10	< 0.01
Praying (< once a day)	0.13	− 0.29 to 0.56	0.04	0.54	0.13	− 0.18 to 0.45	0.06	0.4	− 0.1	− 0.42 to 0.22	− 0.04	0.55
Praying (≥ once a day)	0.002	− 0.54 to 0.55	0.00	1.00	− 0.07	− 0.47 to 0.34	− 0.03	0.75	− 0.54	− **0.95 to** – **0.13**	− **0.23**	**0.01**
Income	0.02	− 0.08 to; 0.12	0.01	0.71	0.03	− 0.05 to 0.11	0.03	0.43	− 0.01	− 0.09 to 0.08	− 0.01	0.9
Having a partner	− 0.37	− 0.87 to 0.14	− 0.11	0.15	− 0.01	− 0.38 to 0.36	− 0.00	0.96	− 0.22	− 0.60 to 0.17	− 0.09	0.27
Mastery	** − 0.07**	− **0.13 to -0.01**	− **0.09**	**0.02**	− 0.02	− **0.06 to 0.03**	− **0.03**	**0.46**	− **0.01**	− 0.06 to 0.03	− 0.02	0.55
Functional limitations	**0.13**	− **0.001 to 0.26**	**0.07**	**0.05**	0.04	− 0.05 to 0.14	0.03	0.4	− 0.07	− 0.16 to 0.03	− 0.05	0.17
Vaccinated	0.17	− 0.29 to 0.62	− 0.05	0.47	0.1	− 0.44 to 0.24	0.04	0.55	0.29	− 0.63 to 0.06	0.12	0.1

Significant results are presented **bold**, p < 0.05

In model 1 we found that each 1-point increase in COVID-19 exposure was associated with an unstandardized increase of 0.03 (CI: 0.00, 0.07; p = 0.07) in depressive symptom score, a 0.04 increase (CI: 0.01,0.07; p < 0.01) in anxiety score and a 0.10 increase (CI: 0.08,0.13; p < 0.01) in loneliness. After adjustment for pre-pandemic mental health (model 2), we found that higher COVID-19 exposure was associated with an (unstandardized) increase of 0.11 (CI: 0.07 – 0.14, p < 0.01) in depressive symptoms, 0.08 increase (CI: 0.06 – 0.10, p < 0.01) in anxiety symptoms and 0.11 increase (CI: 0.09 – 0.13, p < 0.01) in loneliness. Standardized effects indicated that COVID-19 exposure had a larger effect on the increase in loneliness (std. B = 0.30) than on depressive (std. B = 0.20) and anxiety symptoms (std. B = 0.21).

### Main effects of potential protective factors

Main effects from the model including all potential protective factors (model 3) showed that regardless of baseline mental health, internet use and higher mastery were associated with less increase in depressive symptoms, network size was associated with less increase in anxiety symptoms, and female gender, network size and praying were associated with less increase in loneliness.

### Buffering effects of potential protective factors

**Fig. 3:**
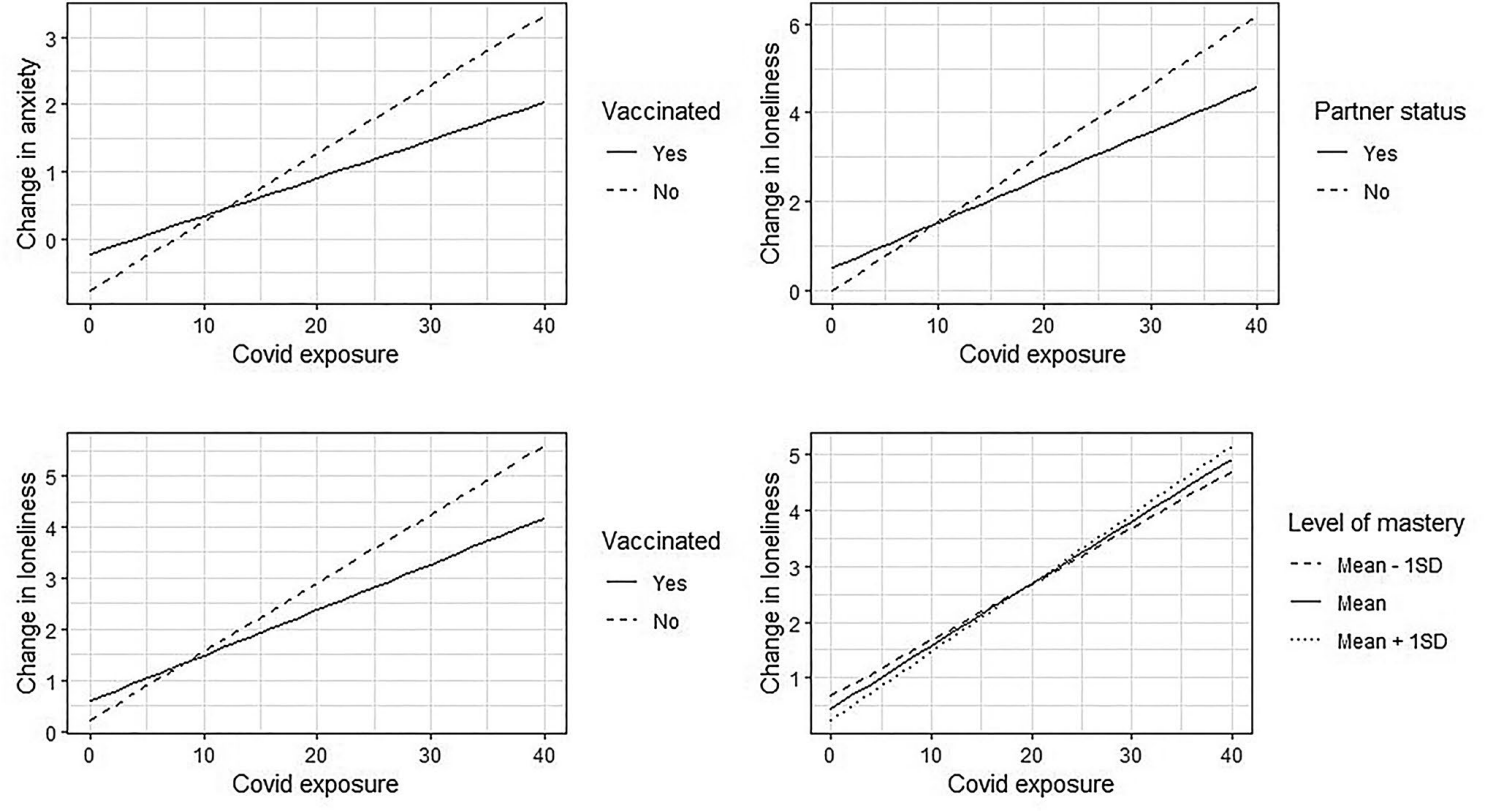
Interaction effects between COVID-19 exposure and protective factors

We found four interaction effects between COVID-19 exposure and potential protective factors with p < 0.10 (Table 3). First, increase in anxiety symptoms associated with more COVID-19 exposure was weaker in persons who were COVID-19 vaccinated (B =  − 0.05 (CI: − 0.09 to 0.00, p < 0.04). Second, increase in loneliness associated with more COVID-19 exposure was also weaker in persons who were vaccinated (B =  − 0.04 (CI: − 0.09 to − 0.01, p < 0.08). Third, the increase in loneliness associated with more COVID-19 exposure was weaker in persons who had a partner (B =  − 0.05 (CI: − 0.10 to 0.01), p = 0.09). Fourth, increase in loneliness associated with more COVID-19 exposure was larger in persons who reported higher mastery (i.e. who felt more personal control over life) (B = 0.01 (CI: 0.00; 0.01), p = 0.06). Visual representations of the interaction effects are shown in Fig. 3. Details on beta, p and CI of main effects and confounding factors are shown in supplementary table 4.

**Table 3:** Buffering effects on the impact of covid-exposure on change in mental health, with p < 0.10

	Anxiety				Loneliness			
	Unst.B	CI	Std. B	p	Unst.B	CI	Std. B	p
With partner status								
Covid-exposure					**0.15**	**0.10 to 0.19**	**0.40**	** < 0.01**
Having a partner					0.24	− 0.61 to 0.12	− 0.27	0.19
Covid-exposure*Partner					− **0.05**	− **0.10 to 0.01**	− **0.13**	**0.09**
With mastery								
Covid-exposure					**0.11**	**0.08 to 0.13**	**0.30**	** < 0.01**
Mastery					− 0.02	− 0.06 to 0.02	− 0.04	0.23
Covid-exposure*Mastery					**0.01**	**0.00 to 0.01**	**0.06**	**0.06**
With vaccination status								
Covid-exposure	**0.10**	**0.07 to 0.13**	**0.26**	** < 0.01**	**0.13**	**0.10 to 0.16**	**0.35**	** < 0.01**
Vaccinated	− 0.11	− 0.46 to 0.24	− 0.04	0.53	− 0.27	− 0.62 to 0.07	− 0.12	0.13
Covid-exposure*Vaccinated	− **0.05**	− **0.09 to** − **0.002**	− **0.12**	**0.04**	− **0.04**	− **0.09 to 0.01**	− **0.02**	**0.08**

Significant results are presented bold, p < 0.10

## Discussion

Our findings showed that depressive and anxiety symptoms and loneliness were persistently higher during the first 1.5 year of the COVID-19 pandemic than before the pandemic in the general older population in the Netherlands. While depressive and anxiety symptoms were higher just after the start of the pandemic and stabilized during the second year of the pandemic, loneliness continued to increase during the second year. These results are consistent with other studies showing average increases in depressive symptoms, anxiety symptoms and loneliness during the pandemic [1, 2, 16]. Furthermore, we found higher cumulative exposure to COVID-19 pandemic-related adversity to be associated with stronger increases in depressive symptoms, anxiety symptoms and loneliness. This indicates that there is substantial heterogeneity in the extent to which the mental health of older adults was affected by the pandemic. Moreover, this indicates a dose–response relationship with cumulative stressors conceivably leading to more mental health symptoms and loneliness. Several factors were associated with less increase of depressive and anxiety symptoms and loneliness but did not buffer the specific effects of COVID-19 exposure on mental health. Being vaccinated buffered the impact of COVID-19 exposure on anxiety and loneliness, and having a partner buffered the impact of COVID–19 exposure on loneliness. Higher mastery appeared to be related to a stronger rather than weaker effect of Covid-19 on loneliness.

### Protective and buffering factors

We deem it hopeful that in addition to protection against the virus, vaccination against COVID-19 also appears to have psychological benefits and help ameliorate the mental health effects of pandemic-related adversity. Given the higher risks of severe illness due to a COVID-19 infection in old age, vaccination may have induced a feeling of relief and increased confidence which may explain its buffering effect of pandemic-related exposure.

Although we observed an average increase in anxious and depressive symptomatology and loneliness across our general population-sample of older adults, this increase was smaller in those with poorer pre-pandemic mental health. This is in line with previous research in psychiatric case–control cohorts, which found that symptoms primarily increased in control groups without affective disorders [2]. One explanation could be that for persons with already poor mental health, further deterioration is less likely. Another possible explanation is that those participants with poor mental health were already more exposed to stressors before the pandemic and learned how to better deal with new pandemic-induced stressors. This potential ability to cope better with stressors due to gathering experience with earlier stressors has been called the ‘steeling hypothesis’ and has been found before to reduce the effect of accumulating negative life events on emotional functioning in old age [35, 36]. Another explanation is that those with more pre-pandemic depressive symptoms were already sensitized; this means that the depressogenic effect of new stressful life events, e.g. the COVID-19 pandemic, declines with the number of depressive episodes already experienced. This kindling phenomenon possibly results from the brain becoming depressed due to earlier episodes and is possibly saturable to further episodes [37, 38].

Several factors were associated with changes in mental health during the pandemic. Internet use was associated with less increase in depressive symptoms which adds evidence that in older adults, using the internet can have a protective influence on the probability to develop depression in older persons. Earlier, online communication with family and friends has been found to prevent clinical depression in physically and cognitively independent older adults [12].

The finding that high mastery was associated with less increase in depressive symptomatology during the pandemic is in line with previous findings that a strong internal locus of control (high mastery) alters the psychological effects of various stressors on depressive symptoms. Older adults maybe have, compared to younger adults, better coping strategies and increase of perceived mastery during the COVID-19 pandemic due to developed strengths in life such as reflection, adaptive use of personal memory, generativity and that public health measures resulted in a more quiet and clear everyday life for older adults [39–41].

In addition, we found that the increase in anxiety and loneliness was smaller in older adults with a larger social network. Here we add evidence that social network, loneliness, anxiety, and depression are interrelated. Both social isolation (of which social network is one of the active ingredients) and loneliness have been found to be robust risk factors for anxiety and depression [42]. A larger network probably increases the chance that you have at least some meaningful contacts.

The finding that older women had less increase in loneliness during the pandemic is fitting in the literature as older men have been perceived to be particularly vulnerable to the effects of loss and social isolation [43] while more resilient women may be better able to find protection from loneliness in social ties outside the marriage better than men.

We found praying/meditating associated with less increase in loneliness. Recent research showed that persuing religiosity and spirituality are protective factors in a model designed for the development of depression during the pandemic [44]. To our knowledge no data are available on the potential protective influence of religiosity and spirituality against loneliness in the COVID-19 pandemic. However, we found no specific buffering effect of praying or meditating, so we may assume that praying/meditating is not specifically protecting against COVID-19 associated stress but was associated with mental health outcomes during the pandemic regardless of COVID-19 exposures. We hypothesize that the experience of a presence of a divine power or a focus on the here and now can compensate the loss of social connectedness.

Finally, we also examined whether included factors could buffer the effects of actual exposure to pandemic-related adversity on mental health. Interestingly, we found COVID-19 vaccination a buffering factor against COVID-19 associated anxiety and loneliness. To our knowledge no previous study, analyzing prolonged COVID-19 exposure and mental health changes has showed a possible buffering effect of COVID-19 vaccination. Also we found that having a partner buffered against loneliness in persons with higher COVID-19 adversity exposure. The finding that the increase in COVID-19 adversity associated loneliness was larger just in persons who reported higher mastery (i.e. who felt more personal control over life) before the pandemic is possibly explained as that those with a higher mastery had more to loose of personal control than those with an already lower prepandemic mastery. Another explanation is that those with already less personal control over life already had been steeled due to earlier stressors [35].

### Strengths and limitations

Our study has several strengths. First, we were able to study mental health during the pandemic with two COVID-19 time points integrated in a pre-existing and ongoing cohort study. Second, rather than assuming that the pandemic is a homogeneous exposure to everyone, we used a cumulative COVID-19 exposure index to assess heterogeneity in individual exposure to COVID-19 adversity. With this cumulative exposure, dose–response relations could be studied. Third, we were able to study the main and buffering effects of a wide variety of potential protective factors. As such, we could study in detail potential mechanisms behind differences between older adults in the impact of the pandemic on their mental health.

Some limitations should also be considered. First, the COVID-19 questionnaire was a postal questionnaire, whereas pre-pandemic questionnaires were administered face-to-face. Therefore, part of the observed changes in outcomes could be due to a ‘mode effect’; this is the effect that may occur due to mixed data collection methods. Unfortunately, we cannot determine the extent to which this occurred. However, the fact that loneliness kept increasing during the pandemic suggests that at least for loneliness, the influence of the mode effect is limited. Second, all measures are self-reported. However, self-report on conditions and diseases has previously shown to be fairly accurate in LASA [45]. Third, only participants that were healthy enough to participate were included. Therefore, severely ill and frail older persons may be underrepresented. This could mean that although we found sufficient variation within the included sample, changes in mental health and the impact of COVID-19 exposure may have been underestimated. Fourth, it is possible that we missed specific buffering factors as our COVID-19 adversity exposure might have been incomplete. A possible reason that our results did not show other buffering factors is that our exposure period to COVID-19 adversity has been not long enough and more vulnerable older persons have been underrepresented in our study; older persons with more resources may have been overrepresented.

Last, the LASA-study is a European study with a relatively low participation of immigrants. Therefore, it is difficult to generalize our findings to immigrant groups and low- and middle-income countries where health care and facilities were less present and vaccination unavailable.

### Implications

Although the overall effect of the pandemic on older persons mental health seems modest, our study has various implications.

The most obvious is that exposure to COVID-19 pandemic-related adversity is heterogeneous and that COVID-19 adversity exposure has a cumulative impact on mental health outcomes. This means that targeted measures aimed at specific COVID-19 adversities and specific groups may reduce the impact of the virus on the mental health of older persons.

With the insight that mainly main effects were important in our study we confirm that many older adults have several resources that enable them to face adversity. However older persons severely affected by the pandemic due to an accumulation of COVID-19 adversities will suffer from mental health consequences, even though they have several resources.

The finding that being vaccinated buffers against COVID-19 adversity induced anxiety and loneliness indicating a positive effect of vaccination on mental health is important considering future pandemics.

Moreover, with our study we are possibly providing important information beyond the specific COVID-19 situation which increases the generalizability of our results. Our findings may provide clues about which individual resources are crucial during disasters and other forced isolation situations such as captivity, dictatorship, warfare, and other collective traumas.

## Conclusions

While we found that the average impact of COVID-19 adversity on the mental health of older persons was moderate, the heterogeneity of exposure to COVID-19 adversity was large and the impact of this exposure was found to be cumulative, indicating a dose response relationship. means that older persons with an accumulation of adversities have a considerable risk of mental health symptoms.

Based on our findings, we believe that improving coping, finding meaning, stimulating existing religious and spiritual resources, reinforcing the social network and stimulating internet use may enable older persons to function better in case of pandemics and other collective stressors. Moreover, in future pandemics, if feasible, swift vaccination can have a positive effect on both physical and mental health.

## Supplementary Information

Below is the link to the electronic supplementary material.

Supplementary file1 (DOCX 15 KB)

Supplementary file2 (DOCX 15 KB)

Supplementary file3 (DOCX 13 KB)

Supplementary file4 (DOCX 27 KB)

## Data Availability

The data that support the findings of this study are available from the first three authors upon reasonable request.
