# Supplementary material for: The impact of COVID-19 related adversity on the course of mental health during the pandemic and the role of protective factors: a longitudinal study among older adults in The Netherlands
Source: Soc Psychiatry Psychiatr Epidemiol. 2023 Mar 25;58(7):1109–20. doi: 10.1007/s00127-023-02457-5 (PMC10039342; doi:10.1007/s00127-023-02457-5)
Supplement: Supplementary file 1 — Supplementary file1 (DOCX 15 KB) [file 127_2023_2457_MOESM1_ESM.docx]

Supplementary table 1: items included in the COVID-19 exposure index

| **Item** | **Cut-off** | **Combined as** |
| --- | --- | --- |
| 1. Tested positive for COVID-19 or probable COVID-19 (told by healthcare professional) | No = 0, Yes = 1 | 0 or 2 |
| 1. Hospital admission / ICU admission because of COVID-19 | No = 0, Yes = 1 | 0 or 2 |
| 1. Partner/parent/child with COVID-19 positive test | No = 0, Yes = 1 | 0, 1 or 2 |
| 1. Partner/parent/child with COVID-19 hospital admission or death | No = 0, Yes = 1 | 0, 1 or 2 |
| 1. Sibling/grandchild/other family member with COVID-19 hospital admission or death | No = 0, Yes = 1 | 0, 1 or 2 |
| 1. Neighbor/friend/other acquaintance with COVID-19 hospital admission or death | No = 0, Yes = 1 | 0, 1 or 2 |
| 1. Respondent has been in quarantine | No = 0, Yes = 1 | 0, 1 or 2 |
| 1. GP visit canceled by GP | No = 0, Yes = 1 | 0, 1 or 2 |
| 1. GP visit  replaced by telephone consultation | No = 0, Yes = 1 | 0, 1 or 2 |
| 1. Respondent canceled/postponed GP visit | No = 0, Yes = 1 | 0, 1 or 2 |
| 1. Specialist outpatient visit canceled by outpatient clinic | No = 0, Yes = 1 | 0, 1 or 2 |
| 1. Specialist outpatient visit replaced by telephone consultation | No = 0, Yes = 1 | 0, 1 or 2 |
| 1. Respondent canceled/postponed specialist outpatient visit | No = 0, Yes = 1 | 0, 1 or 2 |
| 1. Respondent postponed help seeking for physical/psychological complaints because of the COVID situation | No = 0, Yes = 1 | 0, 1 or 2 |
| 1. Providing personal/household care: experience of increased burden during the COVID-19 pandemic | No = 0, Yes = 1 | 0, 1 or 2 |
| 1. Providing personal/household care: more than before the COVID-19 pandemic | No = 0, Yes = 1 | 0, 1 or 2 |
| 1. Decrease in received personal/household care during the COVID-19 pandemic | No = 0, Yes = 1 | 0, 1 or 2 |
| 1. Work situation: lower salary due to the COVID-19 pandemic | No = 0, Yes = 1 | 0, 1 or 2 |
| 1. Difficulties with grocery shopping during the COVID-19 pandemic | No = 0, Sometimes or always = 1 | 0, 1 or 2 |
| 1. Weight loss / weight gain because of COVID-19 pandemic | No = 0, Sometimes or always = 1 | 0, 1 or 2 |
| 1. Less physical activity than before the COVID-19 pandemic | No = 0, Sometimes or always = 1 | 0, 1 or 2 |
| 1. Increased alcohol used during the COVID-19 pandemic | No = 0, Sometimes or always = 1 | 0, 1 or 2 |
| 1. Less social contact with family during the COVID-19 pandemic | No = 0, Yes = 1 | 0, 1 or 2 |
| 1. Less social contact with friends and acquaintances during the COVID-19 pandemic | No = 0, Yes = 1 | 0, 1 or 2 |
| 1. Less social contact with formal relationships during the COVID-19 pandemic | No = 0, Yes = 1 | 0, 1 or 2 |
| 1. Impact of job loss/financial problems of respondent during the COVID-19 pandemic | No impact = 0, Moderate or strong = 1 | 0, 1 or 2 |
| 1. Impact of job loss/financial problems of close relative during the COVID-19 pandemic | No impact = 0, Moderate or strong = 1 | 0, 1 or 2 |
| 1. Impact of cancelation of leisure activities during the COVID-19 pandemic | No impact = 0, Moderate or strong = 1 | 0, 1 or 2 |
| 1. Impact of not being able to visit bars, restaurants and/or shops during the COVID-19 pandemic | No impact = 0, Moderate or strong = 1 | 0, 1 or 2 |
| 1. Impact of experience of illness during the COVID-19 pandemic | No impact = 0, Moderate or strong = 1 | 0, 1 or 2 |
| 1. Impact of death or severe illness of partner or household member during the COVID-19 pandemic | No impact = 0, Moderate or strong = 1 | 0, 1 or 2 |
| 1. Death or severe illness of family member or friend during the COVID-19 pandemic | No impact = 0, Moderate or strong = 1 | 0, 1 or 2 |
| 1. Impact of no contact or less contact with children/grandchildren during the COVID-19 pandemic | No impact = 0, Moderate or strong = 1 | 0, 1 or 2 |
| 1. Impact of no contact or less contact with family/friends during the COVID-19 pandemic | No impact = 0, Moderate or strong = 1 | 0, 1 or 2 |
| 1. Impact of difficulties in obtaining essential medication during the COVID-19 pandemic | No impact = 0, Moderate or strong = 1 | 0, 1 or 2 |
