# Supplementary material for: The impact of COVID-19 related adversity on the course of mental health during the pandemic and the role of protective factors: a longitudinal study among older adults in The Netherlands
Source: Soc Psychiatry Psychiatr Epidemiol. 2023 Mar 25;58(7):1109–20. doi: 10.1007/s00127-023-02457-5 (PMC10039342; doi:10.1007/s00127-023-02457-5)
Supplement: Supplementary file 2 — Supplementary file2 (DOCX 15 KB) [file 127_2023_2457_MOESM2_ESM.docx]

Supplementary table 2. prevalences of all the specific COVID -19 exposures

| **Item** | **Cut-off** | **% Yes or moderate/strong impact** | |
| --- | --- | --- | --- |
|  |  | **Cov-1** | **Cov-2** |
| 1. Tested positive for COVID-19 or probable COVID-19 (told by healthcare professional) | No = 0, Yes = 1 | 2.7 | 6.4 |
| 1. Hospital admission / ICU admission because of COVID-19 | No = 0, Yes = 1 | 0.2 | 0.7 |
| 1. Partner/parent/child with COVID-19 positive test | No = 0, Yes = 1 | 3.7 | 13.2 |
| 1. Partner/parent/child with COVID-19 hospital admission or death | No = 0, Yes = 1 | 1.6 | 0.8 |
| 1. Sibling/grandchild/other family member with COVID-19 hospital admission or death | No = 0, Yes = 1 | 4.7 | 7.3 |
| 1. Neighbor/friend/other acquaintance with COVID-19 hospital admission or death | No = 0, Yes = 1 | 30.7 | 25.4 |
| 1. Respondent has been in quarantine | No = 0, Yes = 1 | 12.5 | 11.6 |
| 1. GP visit canceled by GP | No = 0, Yes = 1 | 10.2 | 2.7 |
| 1. GP visit  replaced by telephone consultation | No = 0, Yes = 1 | 15.2 | 8.2 |
| 1. Respondent canceled/postponed GP visit | No = 0, Yes = 1 | 7.4 | 4.4 |
| 1. Specialist outpatient visit canceled by outpatient clinic | No = 0, Yes = 1 | 24.9 | 6.7 |
| 1. Specialist outpatient visit replaced by telephone consultation | No = 0, Yes = 1 | 21.1 | 16.1 |
| 1. Respondent canceled/postponed specialist outpatient visit | No = 0, Yes = 1 | 7.7 | 3.0 |
| 1. Respondent postponed help seeking for physical/psychological complaints because of the COVID situation | No = 0, Yes = 1 | 8.6 | 8.6 |
| 1. Providing personal/household care: experience of increased burden during the COVID-19 pandemic | No = 0, Yes = 1 | 2.4 | 8.2 |
| 1. Providing personal/household care: more than before the COVID-19 pandemic | No = 0, Yes = 1 | 4.1 | 4.4 |
| 1. Decrease in received personal/household care during the COVID-19 pandemic | No = 0, Yes = 1 | 4.9 | 1.6 |
| 1. Work situation: lower salary due to the COVID-19 pandemic | No = 0, Yes = 1 | 1.6 | 0.4 |
| 1. Difficulties with grocery shopping during the COVID-19 pandemic | No = 0, Sometimes or always = 1 | 15.2 | 14.0 |
| 1. Weight loss / weight gain because of COVID-19 pandemic | No = 0, Sometimes or always = 1 | 36.9 | 44.4 |
| 1. Less physical activity than before the COVID-19 pandemic | No = 0, Sometimes or always = 1 | 50.5 | 50.4 |
| 1. Increased alcohol used during the COVID-19 pandemic | No = 0, Sometimes or always = 1 | 14.2 | 12.5 |
| 1. Less social contact with family during the COVID-19 pandemic | No = 0, Yes = 1 | 38.0 | 41.9 |
| 1. Less social contact with friends and acquaintances during the COVID-19 pandemic | No = 0, Yes = 1 | 41.0 | 44.2 |
| 1. Less social contact with formal relationships during the COVID-19 pandemic | No = 0, Yes = 1 | 12.7 | 9.7 |
| 1. Impact of job loss/financial problems of respondent during the COVID-19 pandemic | No impact = 0, Moderate or strong = 1 | 8.9 | 7.6 |
| 1. Impact of job loss/financial problems of close relative during the COVID-19 pandemic | No impact = 0, Moderate or strong = 1 | 15.3 | 15.4 |
| 1. Impact of cancelation of leisure activities during the COVID-19 pandemic | No impact = 0, Moderate or strong = 1 | 72.0 | 74.2 |
| 1. Impact of not being able to visit bars, restaurants and/or shops during the COVID-19 pandemic | No impact = 0, Moderate or strong = 1 | 73.2 | 82.6 |
| 1. Impact of experience of illness during the COVID-19 pandemic | No impact = 0, Moderate or strong = 1 | 10.3 | 13.2 |
| 1. Impact of death or severe illness of partner or household member during the COVID-19 pandemic | No impact = 0, Moderate or strong = 1 | 7.8 | 10.0 |
| 1. Death or severe illness of family member or friend during the COVID-19 pandemic | No impact = 0, Moderate or strong = 1 | 28.8 | 38.0 |
| 1. Impact of no contact or less contact with children/grandchildren during the COVID-19 pandemic | No impact = 0, Moderate or strong = 1 | 55.5 | 52.5 |
| 1. Impact of no contact or less contact with family/friends during the COVID-19 pandemic | No impact = 0, Moderate or strong = 1 | 68.3 | 71.1 |
| 1. Impact of difficulties in obtaining essential medication during the COVID-19 pandemic | No impact = 0, Moderate or strong = 1 | 5.7 | 4.6 |
