# Supplementary material for: The impact of COVID-19 related adversity on the course of mental health during the pandemic and the role of protective factors: a longitudinal study among older adults in The Netherlands
Source: Soc Psychiatry Psychiatr Epidemiol. 2023 Mar 25;58(7):1109–20. doi: 10.1007/s00127-023-02457-5 (PMC10039342; doi:10.1007/s00127-023-02457-5)
Supplement: Supplementary file 3 — Supplementary file3 (DOCX 13 KB) [file 127_2023_2457_MOESM3_ESM.docx]

Supplementary table 3: Change in mental health during the pandemic (sum scores and change scores)

|  | Depressive symptoms | | | | Anxiety symptoms | | | | Loneliness | | | |
| --- | --- | --- | --- | --- | --- | --- | --- | --- | --- | --- | --- | --- |
| **Cross-sectional score** | N | Median | IQR |  | N | Median | IQR |  | N | Median | IQR |  |
| Before COVID | 899 | 3.0 | 5.0 |  | 899 | 2.0 | 4.0 |  | 898 | 0.0 | 2.0 |  |
| COVID 1 | 881 | 5.0 | 4.0 |  | 880 | 3.0 | 4.0 |  | 887 | 2.0 | 4.0 |  |
| COVID 2 | 893 | 5.0 | 5.0 |  | 892 | 3.0 | 3.0 |  | 893 | 3.0 | 4.0 |  |
| **Change score** | Mean | SD | T | P | Mean | SD | T | P | Mean | SD | T | P |
| Before COVID -> COVID 1 | 1.46 | 3.5 | 12.4 | <0.01 | 0.77 | 2.8 | 8.3 | <0.01 | 1.6 | 2.3 | 20.4 | <0.01 |
| COVID 1 -> COVID 2 | 0.09 | 3.2 | 0.8 | 0.41 | -0.13 | 2.5 | 1.5 | 0.13 | 0.65 | 2.4 | 8 | <0.01 |
| Before COVID -> COVID 2 | 1.53 | 3.3 | 14 | <0.01 | 0.44 | 2.3 | 5.7 | <0.01 | 2.04 | 2.3 | 26.2 | <0.01 |
