# Supplementary material for: The impact of COVID-19 related adversity on the course of mental health during the pandemic and the role of protective factors: a longitudinal study among older adults in The Netherlands
Source: Soc Psychiatry Psychiatr Epidemiol. 2023 Mar 25;58(7):1109–20. doi: 10.1007/s00127-023-02457-5 (PMC10039342; doi:10.1007/s00127-023-02457-5)
Supplement: Supplementary file 4 — Supplementary file4 (DOCX 27 KB) [file 127_2023_2457_MOESM4_ESM.docx]

| **Supplementay table 4**  **Models including interaction terms** | |  |  |  |  |  |
| --- | --- | --- | --- | --- | --- | --- |
|  | **Depression** |  |  | **Anxiety** |  |  |
|  | B | p | CI | B | p | CI |
| **Education** |  |  |  |  |  |  |
| Covid-exposure | **0,11** | **< 0,01** | **0,07 - 0,14** | **0,08** | **< 0,01** | **0,06 - 0,10** |
| Age | **0,06** | **< 0,01** | **0,03 - 0,09** | **0,02** | **0,03** | **0,00 - 0,04** |
| Sex | 0,12 | 0,56 | -0,28 - 0,52 | 0,18 | 0,22 | -0,11 - 0,48 |
| Baseline MH | **-0,41** | **< 0,01** | **-0,46 - -0,35** | **-0,43** | **< 0,01** | **-0,49 - -0,38** |
| Education | -0,01 | 0,75 | -0,07 - 0,05 | -0,01 | 0,77 | -0,05 - 0,04 |
| Interaction | 0,001 | 0,82 | -0,01 - 0,01 | 0,003 | 0,46 | -0,004 -0,01 |
| **Internet Use** |  |  |  |  |  |  |
| Covid-exposure | **0,12** | **< 0,01** | **0,05 - 0,20** | **0,07** | **0,02** | **0,01 - 0,13** |
| Age | **0,05** | **< 0,01** | **0,02 - 0,08** | 0,02 | 0,08 | -0,00 - 0,04 |
| Sex | 0,15 | 0,46 | -0,24 - 0,54 | 0,2 | 0,17 | -0,09 - 0,49 |
| Baseline MH | **-0,42** | **< 0,01** | **-0,47 - -0,36** | **-0,44** | **< 0,01** | **-0,50 - -0,38** |
| Internet Use | **-0,87** | **< 0,01** | **-1,44 - -0,29** | -0,25 | 0,25 | -0,68 - 0,18 |
| Interaction | -0,02 | 0,7 | -0,10 - 0,07 | 0,01 | 0,7 | -0,05 - 0,07 |
| **Network Size** |  |  |  |  |  |  |
| Covid-exposure | **0,11** | **< 0,01** | **0,08 - 0,14** | **0,08** | **< 0,01** | **0,06 - 0,11** |
| Age | **0,06** | **< 0,01** | **0,03 - 0,09** | 0,02 | 0,07 | -0,00 - 0,04 |
| Sex | 0,17 | 0,4 | -0,22 - 0,57 | 0,24 | 0,11 | -0,05 - 0,53 |
| Baseline MH | **-0,42** | **< 0,01** | **-0,47 - -0,36** | **-0,44** | **< 0,01** | **-0,50 - -0,38** |
| Network Size | -0,02 | 0,13 | -0,04 - 0,00 | **-0,02** | **0,03** | **-0,03 - -0,00** |
| Interaction | 0,001 | 0,36 | -0,002-0,004 | -0,001 | 0,39 | -0,003-0,001 |
| **Neuroticism** |  |  |  |  |  |  |
| Covid-exposure | **0,11** | **< 0,01** | **0,08 - 0,14** | **0,08** | **< 0,01** | **0,06 - 0,10** |
| Age | **0,06** | **< 0,01** | **0,04 - 0,09** | 0,02 | 0,09 | -0,00 - 0,04 |
| Sex | 0,05 | 0,79 | -0,33 - 0,44 | 0,14 | 0,33 | -0,14 - 0,42 |
| Baseline MH | **-0,5** | **< 0,01** | **-0,56 - -0,44** | **-0,54** | **< 0,01** | **-0,61 - -0,48** |
| Neuroticism | **0,15** | **< 0,01** | **0,11 - 0,20** | **0,13** | **< 0,01** | **0,10 - 0,17** |
| Interaction | -0,002 | 0,51 | -0,01 - 0,00 | -0,001 | 0,52 | -0,01 - 0,00 |
| **Praying** |  |  |  |  |  |  |
| Covid-exposure | **0,1** | **< 0,01** | **0,05 - 0,15** | **0,07** | **< 0,01** | **0,04 - 0,11** |
| Age | **0,06** | **< 0,01** | **0,03 - 0,09** | **0,02** | **0,02** | **0,00 - 0,04** |
| Sex | 0,12 | 0,57 | -0,28 - 0,51 | 0,19 | 0,21 | -0,11 - 0,49 |
| Baseline MH | **-0,41** | **< 0,01** | **-0,46 - -0,35** | **-0,44** | **< 0,01** | **-0,49 - -0,38** |
| Praying 1 | -0,37 | 0,51 | -1,46 - 0,73 | -0,15 | 0,72 | -0,96 - 0,66 |
| Interaction 1 | 0,03 | 0,38 | -0,78 - 2,01 | 0,02 | 0,52 | -0,03 - 0,07 |
| Praying 2 | 0,62 | 0,37 | -0,04 - 0,1 | 0,04 | 0,94 | -0,99 - 1,08 |
| Interaction 2 | -0,04 | 0,43 | -0,12 - 0,05 | -0,01 | 0,84 | -0,07 - 0,06 |
| **Income** |  |  |  |  |  |  |
| Covid-exposure | **0,11** | **< 0,01** | **0,07 - 0,14** | **0,08** | **< 0,01** | **0,06 - 0,10** |
| Age | **0,06** | **< 0,01** | **0,03 - 0,09** | **0,02** | **0,04** | **0,00 - 0,04** |
| Sex | 0,09 | 0,67 | -0,31 - 0,49 | 0,19 | 0,22 | -0,11 - 0,49 |
| Baseline MH | **-0,41** | **< 0,01** | **-0,47 - -0,36** | **-0,44** | **< 0,01** | **-0,50 - -0,38** |
| Income | -0,05 | 0,32 | -0,14 - 0,05 | -0,01 | 0,67 | -0,08 - 0,05 |
| Interaction | 0,001 | 0,86 | -0,01 - 0,01 | -0,003 | 0,6 | -0,01 - 0,01 |
| **Partner** |  |  |  |  |  |  |
| Covid-exposure | **0,13** | **< 0,01** | **0,06 - 0,19** | **0,1** | **< 0,01** | **0,06 - 0,15** |
| Age | **0,06** | **< 0,01** | **0,03 - 0,09** | **0,02** | **0,03** | **0,00 - 0,04** |
| Sex | 0,08 | 0,71 | -0,33 - 0,48 | 0,19 | 0,23 | -0,12 - 0,49 |
| Baseline MH | **-0,42** | **< 0,01** | **-0,47 - -0,36** | **-0,44** | **< 0,01** | **-0,50 - -0,38** |
| Partner | -0,28 | 0,26 | -0,77 - 0,21 | -0,05 | 0,8 | -0,40 - 0,31 |
| Interaction | -0,02 | 0,55 | -0,10 - 0,05 | 0,03 | 0,26 | -0,09 - 0,02 |
| **Mastery** |  |  |  |  |  |  |
| Covid-exposure | **0,1** | **< 0,01** | **0,07 - 0,13** | **0,08** | **< 0,01** | **0,05 - 0,10** |
| Age | **0,05** | **< 0,01** | **0,02 - 0,08** | 0,02 | 0,13 | -0,01 - 0,04 |
| Sex | 0,07 | 0,73 | -0,32 - 0,46 | 0,17 | 0,27 | -0,13 - 0,46 |
| Baseline MH | **-0,46** | **< 0,01** | **-0,52 - -0,40** | **-0,46** | **< 0,01** | **-0,52 - -0,40** |
| Mastery | **-0,11** | **< 0,01** | **-0,17 - -0,05** | **-0,05** | **0,01** | **-0,10 - -0,01** |
| Interaction | 0,004 | 0,32 | -0,004 - 0,01 | 0,002 | 0,46 | -0,004 - 0,01 |
| **Functional Limitiations** |  |  |  |  |  |  |
| Covid-exposure | **0,1** | **< 0,01** | **0,07 - 0,13** | **0,07** | **< 0,01** | **0,05 - 0,10** |
| Age | **0,05** | **< 0,01** | **0,02 - 0,08** | 0,02 | 0,16 | -0,01 - 0,04 |
| Sex | **0,15** | **0,47** | **-0,25 - 0,54** | 0,2 | 0,18 | -0,09 - 0,49 |
| Baseline MH | **-0,43** | **< 0,01** | **-0,49 - -0,38** | **-0,45** | **< 0,01** | **-0,51 - -0,39** |
| Functional Limitiations | 0,15 | 0,03 | 0,02 - 0,29 | 0,08 | 0,1 | -0,02 - 0,18 |
| Interaction | 0,01 | 0,44 | -0,01 - 0,02 | 0,01 | 0,18 | -0,004 - 0,02 |
| **Vaccinated** |  |  |  |  |  |  |
| Covid-exposure | **0,1** | **0,02** | **0,02 - 0,19** | 0,05 | 0,09 | -0,01 - 0,11 |
| Age | **0,06** | **< 0,01** | **0,03 - 0,09** | **0,02** | **0,05** | **0,00 - 0,05** |
| Sex | 0,13 | 0,51 | -0,26 - 0,52 | 0,2 | 0,18 | -0,09 - 0,49 |
| Baseline MH | **-0,41** | **< 0,01** | **-0,46 - -0,35** | **-0,44** | **< 0,01** | **-0,49 - -0,38** |
| Vaccinated | -0,15 | 0,51 | -0,57 - 0,28 | 0,01 | 0,94 | -0,31 - 0,33 |
| Interaction | 0,003 | 0,92 | -0,05 - 0,05 | 0,02 | 0,37 | -0,02 - 0,06 |

**Supplementary table 4**

**Models including interaction terms**

|  | | | **Loneliness** | |  | |  |
| --- | --- | --- | --- | --- | --- | --- | --- |
|  | | | B | | p | | CI |
| **Education** | | |  | |  | |  |
| Covid-exposure | | | **0,11** | | **< 0,01** | | **0,09 - 0,13** |
| Age | | | **0,03** | | **0,01** | | **0,01 - 0,05** |
| Sex | | | **-0.34** | | **0.02** | | **-0,63 - -0.05** |
| Baseline MH | | | **-0,26** | | **< 0,01** | | **-0,32 - -0,20** |
| Education | | | -0,01 | | 0,66 | | -0,05 - 0,03 |
| Interaction | | | 0,002 | | 0,49 | | -0,004 - 0,01 |
| **Internet Use** | | |  | |  | |  |
| Covid-exposure | | | **0,09** | | **< 0,01** | | **0,03 - 0,15** |
| Age | | | **0,03** | | **0,02** | | **0,00 - 0,05** |
| Sex | | | **-0.32** | | **0,03** | | **-0,61 - 0,04** |
| Baseline MH | | | **-0,26** | | **< 0,01** | | **-0,33 - -0,20** |
| Internet Use | | | 0.11 | | 0.61 | | 0.55 – 0.32 |
| Interaction | | | 0,02 | | 0.47 | | -0,04 - 0,09 |
| **Network Size** | | |  | |  | |  |
| Covid-exposure | | | **0,11** | | **< 0,01** | | **0,09 - 0,14** |
| Age | | | 0.02 | | 0,05 | | -0,00 - 0,04 |
| Sex | | | -0,27 | | 0,07 | | -0,56 - 0,02 |
| Baseline MH | | | **-0,29** | | **< 0,01** | | **-0,36 - 0,23** |
| Network Size | | | **-0,03** | | **<0.01** | | **-0,04 - -0,01** |
| Interaction | | | -0,001 | | 0,32 | | -0,003 -0,001 |
| **Neuroticism** | | |  | |  | |  |
| Covid-exposure | | | **0,11** | | **< 0,01** | | **0,08 - 0,13** |
| Age | | | **0,03** | | **0,01** | | **0,01 - 0,05** |
| Sex | | | **-0.39** | | **0.01** | | **-0,68 - -0,11** |
| Baseline MH | | | **-0,29** | | **< 0,01** | | **-0,35 - -0,22** |
| Neuroticism | | | **0,06** | | **< 0,01** | | **0,02 - 0,09** |
| Interaction | | | -0,003 | | 0,19 | | -0,01 - 0,00 |
| **Praying** | | |  | |  | |  |
| Covid-exposure | | | **0,11** | | **< 0,01** | | **0,08 - 0,15** |
| Age | | | **0,03** | | **< 0,01** | | **0,01 - 0,05** |
| Sex | | | -0.29 | | 0,05 | | -0,58 - 0,00 |
| Baseline MH | | | **-0,26** | | **< 0,01** | | **-0,32 - -0,20** |
| Praying 1 | | | -0,43 | | 0,3 | | -1,24 - 0,38 |
| Interaction 1 | | | 0,02 | | 0,44 | | -0,03 – 0.07 |
| Praying 2 | | | 0,19 | | 0,71 | | -0,84 – 1.23 |
| Interaction 2 | | | -0,05 | | 0,13 | | -0,12 - 0,02 |
| **Income** | | |  | |  | |  |
| Covid-exposure | **0,11** | **< 0,01** | | **0,08 - 0,13** | |  |  |
| Age | **0,02** | **0,03** | | **0,00 - 0,05** | |  |  |
| Sex | **-0.37** | **0,01** | | **-0,67 - -0.08** | |  |  |
| Baseline MH | **-0,27** | **< 0,01** | | **-0,33 - -0,20** | |  |  |
| Income | -0,04 | 0,8 | | -0,01- 0,01 | |  |  |
| Interaction | 0,001 | 0,86 | | -0,01 - 0,01 | |  |  |
| **Partner** |  |  | |  | |  |  |
| Covid-exposure | **0,15** | **< 0,01** | | **0,06 - 0,19** | |  |  |
| Age | **0,02** | **0,03** | | **0,00 - 0,05** | |  |  |
| **Sex** | **-0.39** | **0,01** | | **-0,68 - -0.09** | |  |  |
| Baseline MH | **-0,27** | **< 0,01** | | **-0,34 - -0,21** | |  |  |
| Partner | -0,23 | 0,21 | | -0,60 - 0,13 | |  |  |
| Interaction | -0,05 | 0,1 | | -0,10 - 0,01 | |  |  |
| **Mastery** |  |  | |  | |  |  |
| Covid-exposure | **0,11** | **< 0,01** | | **0,08 - 0,13** | |  |  |
| Age | **0,02** | **0,03** | | **0,00 - 0,05** | |  |  |
| Sex | **-0.35** | **0.02** | | **-0,64 - -0.06** | |  |  |
| Baseline MH | **-0,27** | **< 0,01** | | **-0,33 - -0,20** | |  |  |
| Mastery | -0,02 | 0.25 | | -0,07 - 0,02 | |  |  |
| Interaction | 0,01 | 0,07 | | -0,00 - 0,01 | |  |  |
| **Functional Limitiations** |  |  | |  | |  |  |
| Covid-exposure | **0,11** | **< 0,01** | | **0,09 - 0,13** | |  |  |
| Age | **0,35** | **0,01** | | **0,01 - 0,05** | |  |  |
| Sex | **-0.33** | **0.03** | | **-0,61 - -0.04** | |  |  |
| Baseline MH | **-0,26** | **< 0,01** | | **-0,32 - -0,20** | |  |  |
| Functional Limitiations | -0.01 | 0.82 | | -0,11 - 0,08 | |  |  |
| Interaction | -0,01 | 0,48 | | -0,02 - 0,01 | |  |  |
| **Vaccinated** |  |  | |  | |  |  |
| Covid-exposure | 0.05 | 0,1 | | -0,01 - 0,12 | |  |  |
| Age | **0,04** | **< 0,01** | | **0,01 - 0,06** | |  |  |
| Sex | **-0.31** | **0.03** | | **-0,26 - - 0.03** | |  |  |
| Baseline MH | **-0,27** | **< 0,01** | | **-0,33 - - 0,20** | |  |  |
| Vaccinated | 0.28 | 0,09 | | -0,04 - 0,60 | |  |  |
| Interaction | 0,04 | 0,07 | | -0,003 - 0,08 | |  |  |

Significant results are presented **bold,** p < 0.10
